# Prognostic value of Midkine expression in patients with solid tumors: a systematic review and meta-analysis

**DOI:** 10.18632/oncotarget.23892

**Published:** 2018-01-04

**Authors:** Luo Zhang, Xing Song, Yingjie Shao, Changping Wu, Jingting Jiang

**Affiliations:** ^1^ Department of Tumor Biological Treatment, The Third Affiliated Hospital of Soochow University, Changzhou, Jiangsu Province, China; ^2^ Department of Oncology, The Third Affiliated Hospital of Soochow University, Changzhou, Jiangsu Province, China

**Keywords:** Midkine, prognosis, solid tumor, meta-analysis

## Abstract

**Background:**

Accumulated studies have shown the important role of Midkine (MDK) protein in various solid tumors and indicated its correlation with patients’ survival. This meta-analysis was performed to further explore the prognostic value of MDK expression in solid tumors.

**Materials and Methods:**

We collected the literatures through searching PubMed, Embase and the Cochrane Library (last up to April 10, 2017) to assess the effect of MDK on survival in solid tumor patients. The STATA 12.0 software was used for the meta-analysis. Fixed-effects models or random-effects models were used to estimate the pooled hazard ratios (HRs) for overall survival (OS).

**Results:**

A total of 2097 patients from 17 observational studies were summarized. High expression of MDK was notably associated with worse OS in solid tumor patients. (pooled HR = 1.96; 95% CI = 1.67–2.31). The subgroup analysis of tumor type demonstrated negative impact of elevated MDK on OS in most solid tumor patients (*P* < 0.05), while MDK had no relevance with OS in the patients with OSCC (pooled HR = 1.68; 95% CI = 0.84–3.36; *P =* 0.145) or HNSCC (pooled HR = 1.56; 95% CI = 0.96–2.51; *P =* 0.075).

**Conclusions:**

The present meta-analysis clarifies that MDK is a potential prognostic biomarker in solid tumor patients. Future large-scale prospective clinical trials are needed to determine the prognostic value of MDK in solid tumor patients.

## INTRODUCTION

Midkine also known as MDK or MK is a member of heparin-binding growth factor family. MDK is highly expressed at the mid-gestation period, while its expression is declined or undetectable in adults [1, 2]. MDK is a cytokine with complex functions in the nervous system, inflammation, cancer, tissue protection/repair and so on [3]. Its receptors contain protein tyrosine phosphatase ζ, anaplastic lymphoma kinase, Notch 2, proteoglycans and integrins, low density lipoprotein receptor-related protein (LRP). In tumors, MDK can promote tumor cells differentiation, proliferation, anti-apoptosis, chemoresistance, transformation and epithelial-mesenchymal transition (EMT) [4–7]. High MDK protein expression has been reported in several cancer types and associated with the cancer progression, including gastric cancer [8], pancreatic cancer [9], lung cancer [10], breast cancer [11], colorectal carcinoma [12], esophageal cancer [13], hepatocellular carcinoma [14] and bladder cancer [15]. An important example, MDK is overexpressed in about 50% of pancreatic cancer patients, and participates in the tumor cells chemotherapy resistance through the Notch 2 signaling pathway [16].

The results of numerous clinical trials have suggested that high MDK expression is associated with shorter overall survival (OS) in various types of cancers. However, the reliability and degree of the prognostic effect of MDK in solid tumors has not yet been systematically evaluated. Hence, we performed a systematic review and meta-analysis to assess the prognostic value of elevated MDK in solid tumors. It was hypothesized that MDK could serve as a biomarker of poor prognosis in patients with solid tumors.

## RESULTS

### Study characteristics

**Figure 1:**
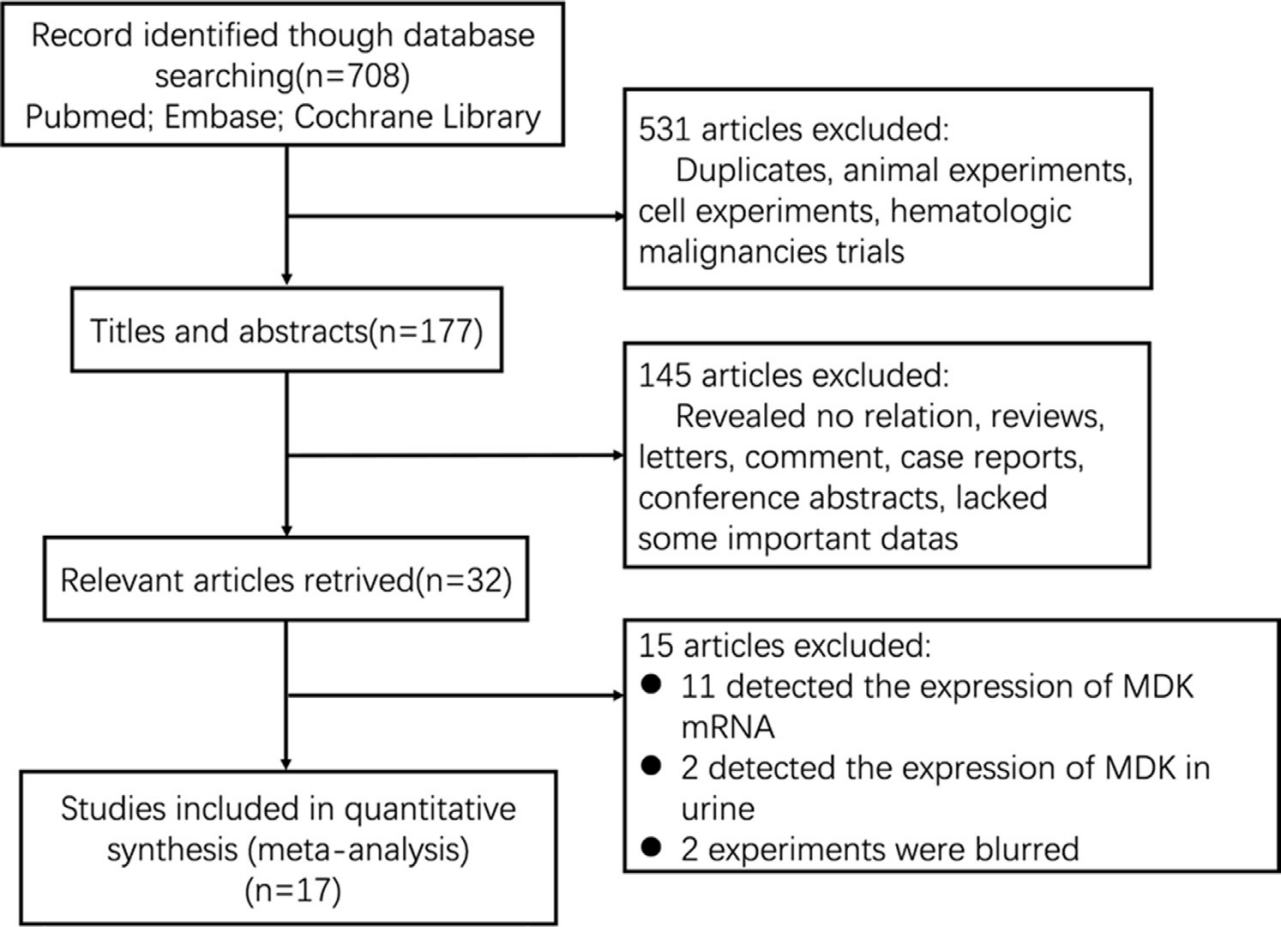
Flow diagram of the study selection process

After searching the electronic database, 708 references were primarily shown. After screening the titles, abstracts, and full text of every paper, 32 articles studied the effort of MDK expression on patient survival in multiple malignant tumors were selected (Figure 1). Among these, 15 articles were excluded (eleven detected the expression of MDK mRNA in cancer patients, two detected the expression of MDK in urine, two experiments were blurred). Finally, 17 studies were enrolled into this meta-analysis (Supplementary Table 1) [8–11, 17–29]. Supplementary Table 1 shows the main information of the included studies. A total of 2097 patients from China, Japan, and Germany were diagnosed with multiple cancers, including breast cancer, gastric cancer, gastrointestinal stromal tumor (GIST), glioma, head and neck squamous cell carcinoma (HNSCC), oral squamous cell carcinoma (OSCC), non-small cell lung cancer (NSCLC), esophageal squamous cell carcinoma (ESCC), pancreatic cancer, malignant mesothelioma (MM), or neuroblastoma. Nine studies (56%) reported on Chinese, seven studies (38%) on Japanese and only one study (6%) on German. The endpoint OS was addressed in 17 studies. HRs were provided directly in 9 studies and estimated from Kaplan-Meier Survival curves in the other 8 studies. The MDK protein was detected by immunohistochemistry (IHC) in 11 studies and detected by enzyme-linked immune absorbent assay (ELISA) in the other 6 studies. And these studies reported different cut-off values. The score of all studies varied from 6 to 8 according to the Newcastle-Ottawa Quality Assessment Scale (NOS), with a mean of 7.3. Thus, all studies were eligible for the analysis.

### Overall survival

**Figure 2:**
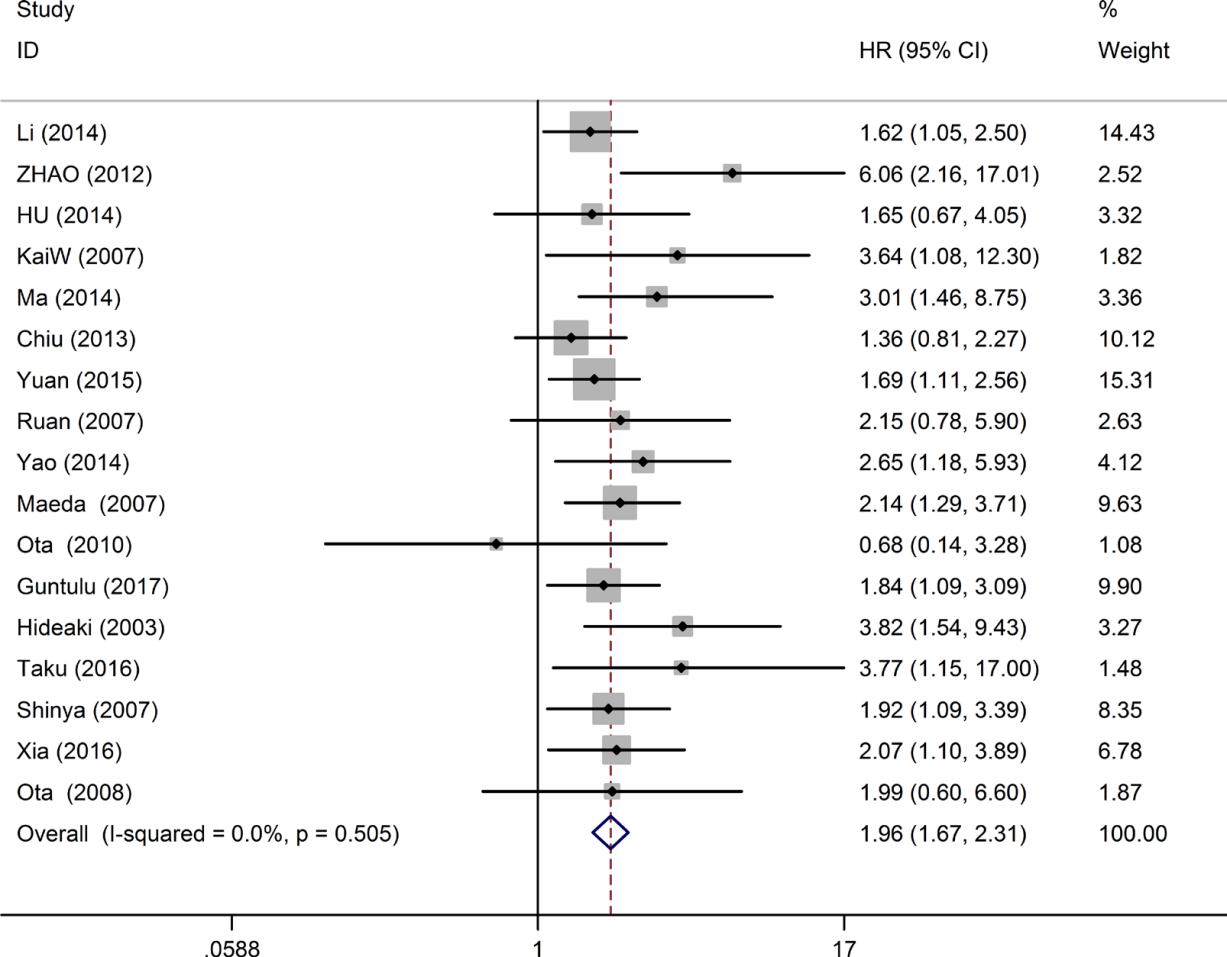
Forest plots of studies evaluating hazard ratios of high MDK expression in solid cancers for overall survival

**Figure 3:**
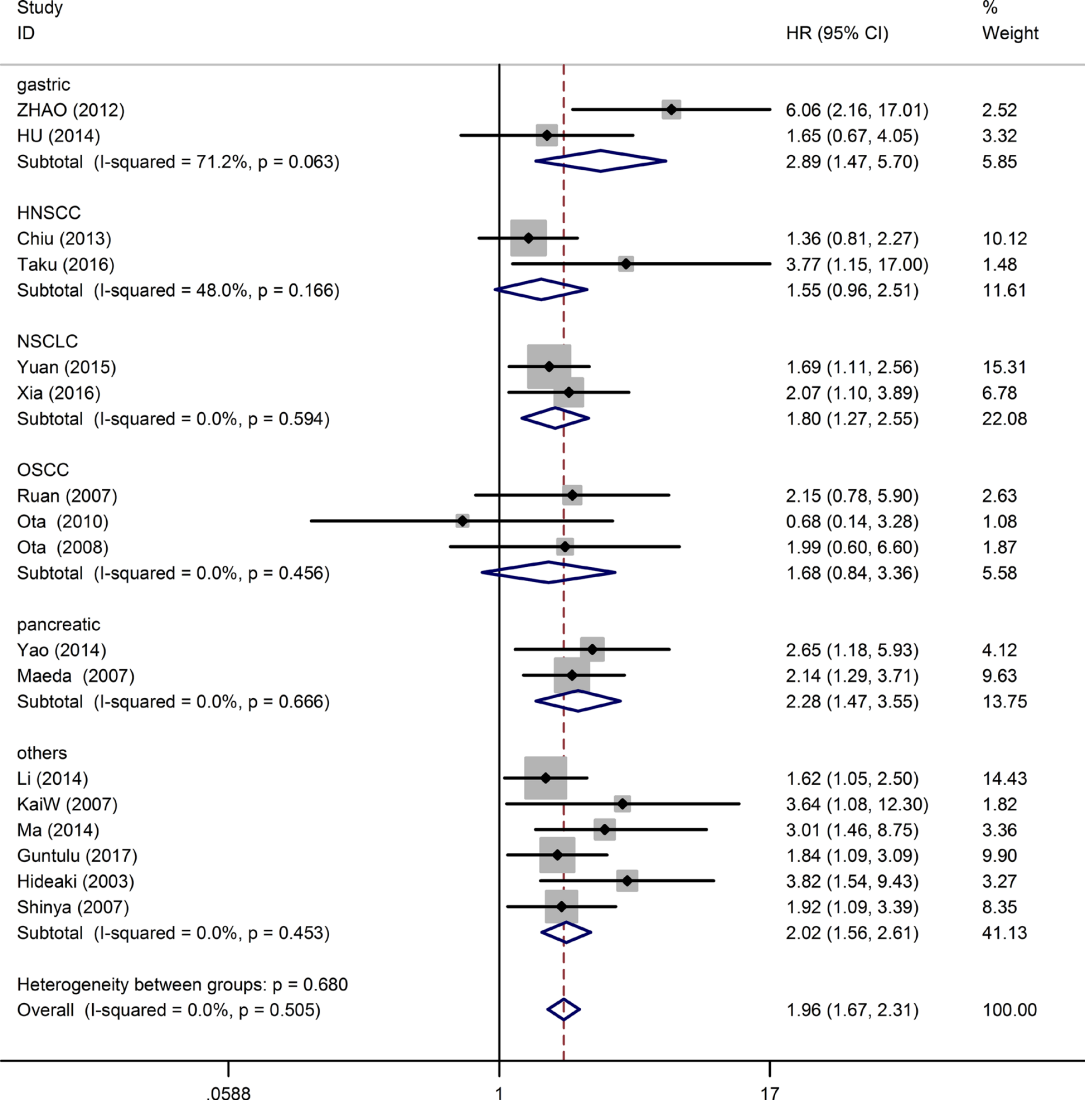
Forest plot of the relationship between high MDK expression and overall survival in patients with a variety of cancers

17 studies consisting of 2097 patients, provided suitable information for OS analysis. The key outcomes of this meta-analysis are shown in Table 1. As there was no problem of heterogeneity in the evaluating OS (I^2^ = 0.0%, *P* = 0.505), a fixed-effect model was used to pool the hazard ratio (HR) and 95% confidence interval (CI). Overall, the pooled analysis demonstrated that high MDK expression was obviously associated with shorter OS in solid tumor patients (pooled HR = 1.96; 95% CI = 1.67–2.31; *P* < 0.001). The study-specific HRs for OS forest plot is presented in Figure 2. Pooled HRs for OS of different cancer types are shown in Figure 3. High expression of MDK was significantly associated with shorter OS in Chinese patients (pooled HR = 1.87; 95% CI = 1.52–2.29; *P* < 0.001), Japanese patients (pooled HR = 2.08; 95% CI = 1.58–2.74; *P* < 0.001) and German patients (pooled HR = 3.64; 95% CI = 1.08–12.28; *P* = 0.037). The subgroup analysis of tumor type demonstrated negative impact of elevated MDK on OS in patients with pancreatic cancer (pooled HR = 2.28; 95% CI = 1.47–3.55; *P* < 0.001), gastric cancer (pooled HR = 2.89; 95% CI = 1.47–5.70; *P* = 0.002), NSCLC (pooled HR = 1.78; 95% CI = 1.27–2.55; *P* = 0.001), and other cancers (pooled HR = 2.20; 95% CI = 1.56–2.61; *P* < 0.001), while MDK had no relevance with OS in the patients with OSCC (pooled HR = 1.68; 95% CI = 0.84–3.36; *P* = 0.145) or HNSCC (pooled HR = 1.56; 95% CI = 0.96–2.51; *P* = 0.075). For OS, pooled HR values > 1 were consistently calculated in subgroup meta-analysis stratified by patients’ nationality, pathologic type, case number, detected method, analysis type and HR obtained method (Table 1).

**Table 1:** Pooled hazard ratios for OS according to subgroup analyses

Outcome subgroup	No. of patients	No. of studies	Fixed-effects model		Heterogeneity	
			HR (95% CI)	*P* value	*I*^2^ (%)	*P*
Overall survival	2097	17	1.96 (1.67,2.31)	< 0.001	0	0.505
Contry						
China	1061	9	1.87 (1.52,2.29)	< 0.001	12.6	0.329
Japan	981	7	2.08 (1.58,2.74)	< 0.001	0	0.581
Germany	55	1	3.64 (1.08,12.28)	0.037	-	-
Detected method						
IHC	1174	11	1.89 (1.55,2.30)	< 0.001	16.9	0.283
ELISA	923	6	2.14 (1.60,2.86)	< 0.001	0	0.740
Case number						
< 100	647	9	2.14 (1.64,2.80)	< 0.001	0	0.729
≥ 100	1450	8	1.86 (1.51,2.29)	< 0.001	25.1	0.229
Pathologic type						
adenocarcinoma	466	5	2.06 (1.55,2.73)	< 0.001	33.3	0.199
Squamous cell carcinoma	555	6	1.83 (1.27,2.63)	0.004	23.6	0.257
Others	1076	6	1.95 (1.53,2.49)	< 0.001	0	0.782
Analysis type						
Multivariate	905	7	1.99 (1.59,2.49)	< 0.001	0	0.516
Univariate	1192	10	1.93 (1.51,2.46)	< 0.001	10.1	0.350
HR obtain method						
Reported in text	1055	9	2.00 (1.63,2.45)	< 0.001	0	0.619
Data extrapolated	1042	8	1.89 (1.43,2.50)	< 0.001	21.5	0.259
Tumor type						
Pancreatic	117	2	2.28 (1.47,3.55)	< 0.001	0	0.666
OSCC	205	3	1.68 (0.84,3.36)	0.145	0	0.456
Gastric	179	2	2.89 (1.47,5.70)	0.002	71.2	0.062
HNSCC	247	2	1.56 (0.96,2.51)	0.075	48	0.166
NSCLC	296	2	1.78 (1.27,2.55)	0.001	0	0.594
Others	1043	6	2.02 (1.56,2.61)	< 0.001	0	0.453

**Figure 4:**
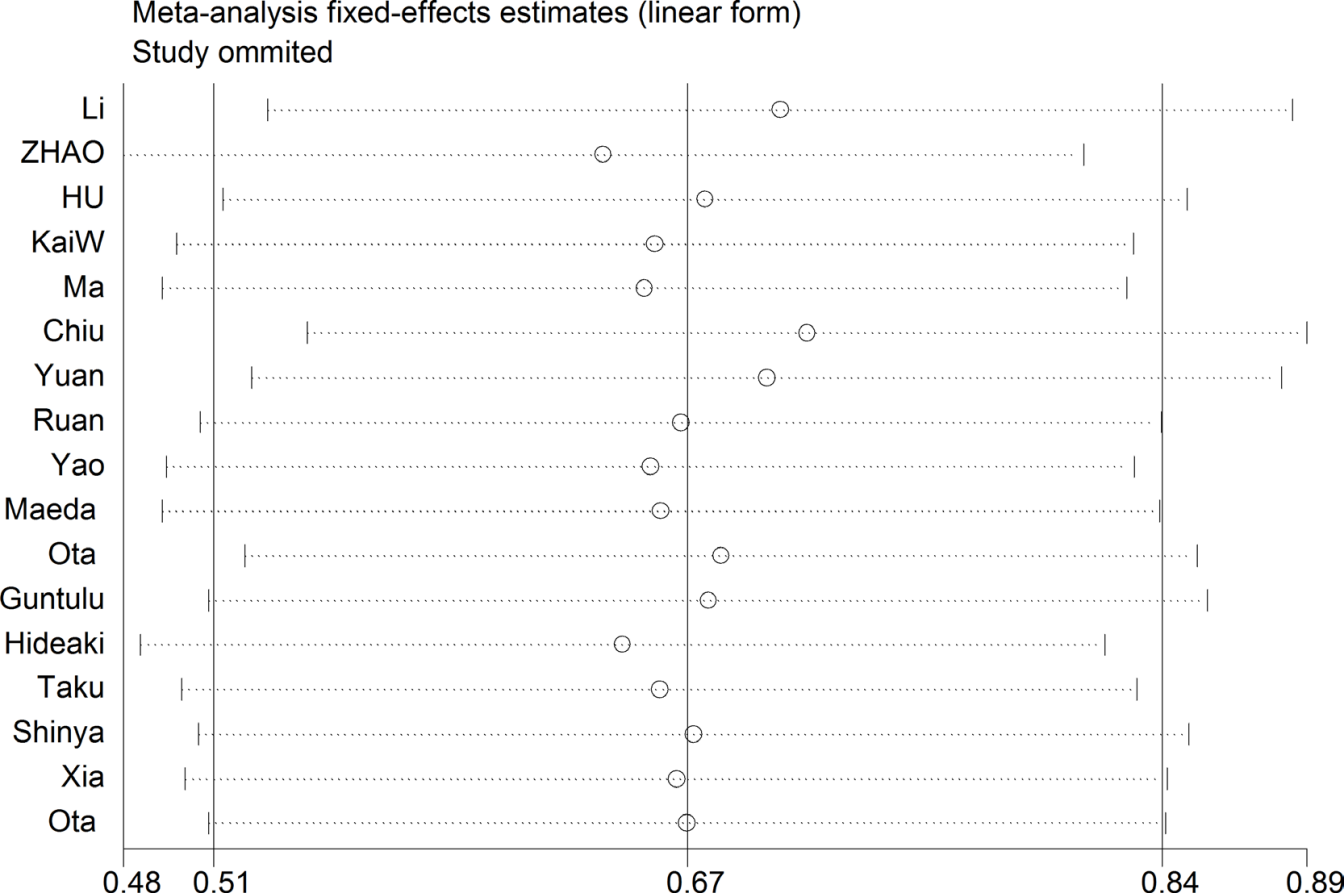
Sensitivity analysis on the relationships between MDK expression and overall survival in solid cancer patients

**Figure 5:**
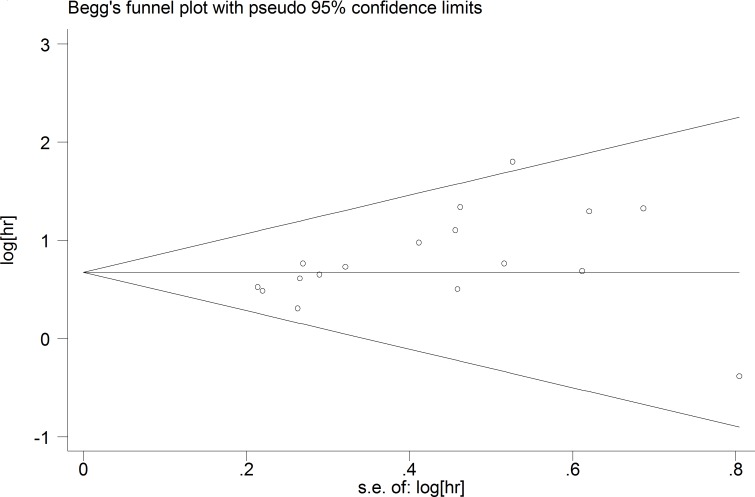
Funnel plots of publication biases on the relationships between MDK expression and overall survival in solid cancer patients

A fixed-effect model was used to perform sensitivity analysis by sequential omission of individual studies, and the result was unaffected by any single study (Figure 4). A meta-regression was conducted to detect the potential factors accountable for the heterogeneity. We used the Funnel plots, Egger’s test and Begg’s test to assess the publication bias of all enrolled studies. Visual observation of the funnel plots (Figure 5) indicated obvious publication bias. Egger’s test and Begg’ tests provided the same result (*P* = 0.036) and (*P* = 0.045). To adjust for publication bias, the ‘‘Trim and Fill” method was used under the fixed-effect model, which calculated corrected pooled multivariable-adjusted HR for OS was 1.81 (95% CI = 1.55–2.12).

## DISCUSSION

MDK is generally highly expressed in diverse malignant solid tumors, and enhances the tumor cells survival, migration, tumor angiogenesis and chemotherapy resistance. As far as we know, this is the first meta-analysis to provide strong evidence that elevated MDK expression is obviously correlated with a worse prognosis in patients with solid tumors. In the subsequent subgroup analyses, the poor prognostic effect of high MDK expression remained stable in different nationality backgrounds. When it came to tumor type, high expression of MDK was significantly related to shorter OS in patients with pancreatic cancer, gastric cancer, NSCLC and other cancers, while MDK had no relevance with OS in the patients with OSCC or HNSCC. In addition to MDK protein, high expression of MDK mRNA may also predict worse OS in patients with cancers [30–32], but the studies were fewer. Serum and plasma levels of MDK are evidently correlated with diagnosis and prognosis in cancer patients (higher MDK level usually suggests tumor occurrence and worse prognosis). Furthermore, MDK was detected in urine, and its high expression in urine might be correlated with worse prognosis in patients with bladder cancer [15, 33]. Moreover, MDK was associated with relapse free survival in patients with cancers [34, 35]. Thus, targeting MDK has been considered to be a promising strategy for cancer therapy. A novel small molecule compound (iMDK) targeted MDK demonstrated by Hao could suppress the growth of H441 cells by inhibiting the PI3K pathway and significantly inhibit tumor growth in a mouse model [36]. Takei established a therapy strategy combining anti-MDK with PTX and found the combination therapy was significative, particularly effective for suppression of angiogenesis [37].

However, there are some deficiencies in this paper as well. First, this study enrolled only 17 eligible studies, which resulted to insufficient data in the subgroup analyses. Publication bias may have been inevitable as studies are more likely to be published if they have positive results than if they have negative results. Second, because the cut-off values for MDK expression were not unified in these studies, different cut-off values might impact on the availability of the prognostic role of MDK in solid tumors. In order to establish the most appropriate cut-off value, further studies with larger sample size are still needed to carry out. In the 17 studies enrolled, 11 studies measured MDK by IHC, while the other 6 studies by ELISA. Compared with other measuring methods, IHC is more economic and easier to be spread for the detection of MDK in tumor tissues. Meanwhile elevated serum level of MDK is independently associated with a poor prognosis of solid tumor patients. So MDK can be detected in serum by ELISA as well. Third, HRs and 95% CIs of several studies were figured out from the data extracted from the survival curves, which inevitably brought about small statistical errors. Fourth, compared to multivariate analyses, univariate analyses may overestimate the effect. However, in our study, compared to the multivariate analyses, univariate analyses based on unadjusted HRs did not reveal an obvious difference in the pooled estimate. Finally, funnel plot graphics showed publication bias. Nevertheless, we found the corrected pooled effect size remained statistically significant after adjusting the publication bias by the ‘‘Trim and Fill” method, which confirmed the reliability of our results.

In conclusion, our results suggest that MDK is a potential biomarker and accurate prognostic predictors in patients with solid tumors. Furthermore, MDK has been considered to be a promising target for the therapy of many kinds of solid tumors. In consideration of the limitation of present paper, this conclusion should be regarded cautiously. In the future, further well-designed, prospective, national multi-center, large sample researches are needed to verify the prognostic value of MDK in solid tumor patients.

## MATERIALS AND METHODS

We carried out this meta-analysis according to the Systematic Reviews and Meta Analyses (PRISMA) guidelines [38].

### Literature search

We searched the literatures through PubMed, Embase and the Cochrane Library to retrieve possible articles relevant to the theme up to April 10, 2017. The key terms used in the search strategy were ‘‘Midkine OR MDK OR MK OR NEGF2’’ (all fields) AND ‘‘tumour OR tumor OR cancer OR neoplasm OR carcinoma’’ (all fields) AND ‘‘survival OR prognostic OR prognosis OR outcome’’ (all fields). The search was limited to clinical trials. We browsed not only the titles and abstracts but also the full texts of identified articles. The availability evaluation and database search was conducted independently by two investigators (L. Zhang and X. Song).

### Inclusion and exclusion criteria

Literatures eligible for this meta-analysis should fulfill the following criteria: (1) all patients must have been diagnosed as solid tumors by pathological examination; (2) the expression level of MDK protein in cancer patients must be analyzed; (3) investigation of the prognostic effect of MDK expression in patients with solid tumors; (4) the HR and 95% CI could be extracted. A lot of literatures were excluded according to the following criteria: (1) hematological malignancies, because the pathogenesis and progress mechanism of hematological malignancies are different from solid tumors; (2) reviews, letters, case reports, conference abstracts, experiments *in vitro* and animal trials; (3) only the latest or complete study was selected if one patient cohort were researched by multiple studies. Two reviewers screened titles, abstracts and the full texts of the articles to choose the eligible publications independently and excluded those articles irrelevant. Different opinions from two investigators were resolved by consultation.

### Data collection and quality assessment

The two researchers collected relevant information from all eligible studies independently, including first author’s surname, year of publication, nationality, tumor type, case number, tumor stage, follow-up months, detected method, the cut-off value, and HR as well as corresponding 95% CI. If multivariate and univariate results were both reported in a study, only the former was selected. The Newcastle-Ottawa Scale (NOS) [39] was used to evaluate the quality of the studies included and a study with a score ≥ 6 was rated as high quality.

### Statistical analysis

The main outcome was OS, comparing cancer patients with high expression of MDK to those with low expression of MDK. The value of MDK expression on prognosis was measured by HRs and 95% CIs. If HRs and 95% CIs were reported in the study, we extracted them directly. Otherwise, they were calculated from available extracted data from Kaplan-Meier survival curves using Engauge Digitizer version 4.1 [40]. Three investigators participated in this process independently to reduce reading variability. We also contact to the authors of related articles if needed. An obtained HR greater than 1 suggested a shorter survival for the patient with elevated MDK expression, while HR less than 1 indicated a longer survival. The I^2^ statistic and Chi-square test (*P* value) were used to assess the statistical heterogeneity [41, 42]. If *P* ≤ 0.05 or I^2^ ≥ 50%, indicating a problem with heterogeneity, we used the random-effects model to calculate pooled HRs. Otherwise, we used the fixed-effects model. Subgroup analyses and meta-regression were performed to further explore the source of identified heterogeneity. Funnel plot, Egger’s test and Begg’s test were carried out to evaluate the publication bias. If publication bias existed, we used the Duval and Tweedie trim-and-fill method to adjust for the effect [43]. STATA 12.0 Software (Stata Corporation, College Station, TX, USA) was used for all analyses, and *P* < 0.05 was considered statistically significant unless otherwise specified.

## SUPPLEMENTARY MATERIALS TABLE




