# Supplementary material for: Prognostic value of Midkine expression in patients with solid tumors: a systematic review and meta-analysis
Source: Oncotarget. 2018 Jan 4;9(37):24821–9. doi: 10.18632/oncotarget.23892 (PMC5973861; doi:10.18632/oncotarget.23892)
Supplement: Supplementary file 1 [file oncotarget-09-24821-s001.pdf]

Prognostic value of Midkine expression in patients with solid tumors: A systematic review and meta-analysis

SUPPLEMENTARY MATERIALS

Supplementary Table 1: Main characteristics of all studies included in the meta-analysis. See_ Supplementary_Table 1.
