# Supplementary material for: Prognostic value of Midkine expression in patients with solid tumors: a systematic review and meta-analysis
Source: Oncotarget. 2018 Jan 4;9(37):24821–9. doi: 10.18632/oncotarget.23892 (PMC5973861; doi:10.18632/oncotarget.23892)
Supplement: Supplementary file 2 [file oncotarget-09-24821-s002.docx]

**Table 1**

**Main characteristics of all studies included in the meta-analysis.**

| First author [References] | Year | Cites core | Country | Cancer | Case number | Tumor stage (I/II/III/IV) | Follow-up (months) | Highexpression n (%) | Detected method | Cut-off value | Multivariate analysis | HRs provided from | Outcome measures | NOS score |
| --- | --- | --- | --- | --- | --- | --- | --- | --- | --- | --- | --- | --- | --- | --- |
| Li[11] | 2014 | 3.65 | China | breast | 170 | 62/108(I–II/III–IV) | Over60 | 95(55.9%) | IHC | score≥6 | yes | Report | OS | 8 |
| Zhao[8] | 2012 | 1.69 | China | gastric | 107 | 30/77(I–II/III–IV) | Over60 | 74(69.2%) | IHC | score>2 | no | SC | OS | 8 |
| Hu[17] | 2014 | 1.69 | China | gastric | 72 | NR | 50 | 55(76.4%) | IHC | ≥10% of cells stained | no | SC | OS | 7 |
| Kaifi[18] | 2007 | 3.50 | Germany | GIST | 55 | NR | Median40 | 31(56%) | IHC | NR | no | Report | OS | 6 |
| Ma[19] | 2014 | 1.56 | China | glioma | 168 | 17/38/63/26(I/II/III/IV) | Over60 | 116(69.0%) | IHC | score≥4 | yes | Report | OS | 8 |
| Chiu[20] | 2013 | 2.01 | China | HNSCC | 144 | 54/90(I-III/IV) | Over60 | 76(52.8%) | IHC | score≥4 | no | SC | OS | 7 |
| Yuan[10] | 2015 | 2.72 | China | NSCLC | 186 | 68/118(I–II/III–IV) | 60 | 114(61.3%) | IHC | score≥6 | yes | Report | OS | 7 |
| Ruan[21] | 2007 | 1.92 | China | OSCC | 62 | 33/29(I–II/III–IV) | 60 | 32(51.6%) | IHC | ≥25% of cells stained | no | SC | OS | 8 |
| Ota[22] | 2010 | 3.08 | Japan | OSCC | 93 | NR | 60 | 62(66.7%) | IHC | Score>2 | no | SC | OS | 7 |
| Yao[9] | 2014 | 3.37 | China | pancreatic | 42 | NR | 42 | 26（61.0%） | IHC | ≥10% of cells stained | no | SC | OS | 7 |
| Maeda[23] | 2007 | 6.18 | Japan | pancreatic | 75 | 65/10(I–II/III–IV) | Median20 | 40(53.5%) | IHC | ≥10% of cells stained | yes | Report | OS | 7 |
| Guntulu[24] | 2017 | 3.27 | Japan | MM | 95 | 12/11/34/36(I/II/III/IV) | Over60 | NR | ELISA | ≥421 pg/ml | no | Report | OS | 7 |
| Hideaki[25] | 2003 | 3.97 | Japan | ESCC | 93 | NR | Median36 | 57(61.3%) | ELISA | ≥300 pg/ml | yes | Report | OS | 7 |
| Taku[26] | 2016 | 3.36 | Japan | HNSCC | 103 | 29/74(I–II/III–IV) | Over60 | 60(58.3%) | ELISA | >482 pg/ml | yes | Report | OS | 7 |
| Shinya[27] | 2007 | 3.97 | Japan | neuroblastoma | 462 | NR | 50 | 276(59.7%) | ELISA | >900 pg/ml | no | SC | OS | 7 |
| Xia[28] | 2016 | 5.17 | China | NSCLC | 110 | 65/10(0–II/IIIa) | 60 | 79(71.8%) | ELISA | >400 pg/ml | yes | Report | OS | 8 |
| Ota[29] | 2008 | 6.18 | Japan | OSCC | 60 | 7/17/7/29(I/II/III/IV) | 60 | 40(66.7%) | ELISA | >650 pg/ml | no | SC | OS | 8 |

gastrointestinal stromal tumor (GIST); glioma, head and neck squamous cell carcinoma (HNSCC); oral squamous cell carcinoma (OSCC); non-small cell lung cancer (NSCLC); esophageal squamous cell carcinoma (ESCC); ELISA: enzyme-linked immunoabsorbent assay; IHC: immunohistochemistry; OS = overall survival; SC: survival curve; NOS = Newcastle-Ottawa Scale
